# Assessing the pre-implementation context for financial navigation in rural and non-rural oncology clinics

**DOI:** 10.3389/frhs.2023.1148887

**Published:** 2023-10-23

**Authors:** Victoria M. Petermann, Caitlin B. Biddell, Arrianna Marie Planey, Lisa P. Spees, Donald L. Rosenstein, Michelle Manning, Mindy Gellin, Neda Padilla, Cleo A. Samuel-Ryals, Sarah A. Birken, Katherine Reeder-Hayes, Allison M. Deal, Kendrel Cabarrus, Ronny A. Bell, Carla Strom, Tiffany H. Young, Sherry King, Brian Leutner, Derek Vestal, Stephanie B. Wheeler

**Affiliations:** ^1^ School of Nursing, University of North Carolina at Chapel Hill, Chapel Hill, NC, United States; ^2^ Lineberger Comprehensive Cancer Center, University of North Carolina at Chapel Hill, Chapel Hill, NC, United States; ^3^ Department of Health Policy and Management, Gillings School of Global Public Health, University of North Carolina at Chapel Hill, Chapel Hill, NC, United States; ^4^ Department of Psychiatry, School of Medicine, University of North Carolina at Chapel Hill, Chapel Hill, NC, United States; ^5^ Department of Medicine, School of Medicine, University of North Carolina at Chapel Hill, Chapel Hill, NC, United States; ^6^ Wake Forest School of Medicine, Wake Forest University, Winston-Salem, NC, United States; ^7^ Wake Forest Baptist Comprehensive Cancer Center, Wake Forest University, Winston-Salem, NC, United States; ^8^ Division of Oncology, University of North Carolina at Chapel Hill, Chapel Hill, NC, United States; ^9^ Buddy Kemp Support Center, Novant Health Cancer Institute, Charlotte, NC, United States; ^10^ Carteret Health Care Cancer Center, Carteret, NC, United States; ^11^ Pardee UNC Health Care, Hendersonville, NC, United States; ^12^ UNC Lenoir Health Care, Kinston, NC, United States

**Keywords:** cancer, financial toxicity, financial navigation, implementation science, rural, organizational readiness framework for advancing implementation science

## Abstract

**Background:**

Financial navigation (FN) is an evidence-based intervention designed to address financial toxicity for cancer patients. FN's success depends on organizations' readiness to implement and other factors that may hinder or support implementation. Tailored implementation strategies can support practice change but must be matched to the implementation context. We assessed perceptions of readiness and perceived barriers and facilitators to successful implementation among staff at nine cancer care organizations (5 rural, 4 non-rural) recruited to participate in the scale-up of a FN intervention. To understand differences in the pre-implementation context and inform modifications to implementation strategies, we compared findings between rural and non-rural organizations.

**Methods:**

We conducted surveys (*n* = 78) and in-depth interviews (*n* = 73) with staff at each organization. We assessed perceptions of readiness using the Organizational Readiness for Implementing Change (ORIC) scale. In-depth interviews elicited perceived barriers and facilitators to implementing FN in each context. We used descriptive statistics to analyze ORIC results and deductive thematic analysis, employing a codebook guided by the Consolidated Framework for Implementation Research (CFIR), to synthesize themes in barriers and facilitators across sites, and by rurality.

**Results:**

Results from the ORIC scale indicated strong perceptions of organizational readiness across all sites. Staff from rural areas reported greater confidence in their ability to manage the politics of change (87% rural, 76% non-rural) and in their organization's ability to support staff adjusting to the change (96% rural, 75% non-rural). Staff at both rural and non-rural sites highlighted factors reflective of the Intervention Characteristics (relative advantage) and Implementation Climate (compatibility and tension for change) domains as facilitators. Although few barriers to implementation were reported, differences arose between rural and non-rural sites in these perceived barriers, with non-rural staff more often raising concerns about resistance to change and compatibility with existing work processes and rural staff more often raising concerns about competing time demands and limited resources.

**Conclusions:**

Staff across both rural and non-rural settings identified few, but different, barriers to implementing a novel FN intervention that they perceived as important and responsive to patients' needs. These findings can inform how strategies are tailored to support FN in diverse oncology practices.

## Introduction

Financial toxicity (FT) is the multidimensional side effect of the continually increasing financial burden of accessing and receiving cancer treatment due to the rising costs of cancer care in the United States (1, 2). FT can result from direct medical and non-medical costs associated with accessing treatment (i.e., copays, prescriptions, transportation etc.) and indirect costs of illness (i.e., productivity loss and forgone income). The consequences of this financial hardship include health and non-health crises, ranging from diminished health-related quality of life to increased risk of bankruptcy and increased risk of mortality (3–6). These elevated risks can persist for years after patients complete cancer treatment (7). Rural oncology patients, in particular, are at greater risk for experiencing FT, since rural communities have a disproportionate share of older patients living on fixed incomes and are more often designated medically underserved areas (8–10). Addressing the financial needs of oncology patients is central to ensuring access to equitable cancer care throughout treatment and survivorship in both rural and non-rural communities (11).

Although policy- and system-level reforms are needed to reduce the direct medical cost of care to patients, additional work is necessary at the provider- and health care organization-levels to meet patients' immediate financial needs (12). Financial navigation (FN) is an evidence-based intervention that can help reduce the burden of cancer care costs and address FT in oncology patients by systematically assessing financial needs through comprehensive screening and intake assessment and addressing those needs through resource connection and application assistance, with ongoing monitoring and follow-up (13–15). Cancer navigators and financial counselors may often take on aspects of financial navigation, usually in the absence of a financial navigator presence at a cancer center, but their roles are, respectively, more focused on helping patients navigate treatment and helping facilitate payment to a hospital or health care system. Financial navigators are unique in that they are trained to (1) help connect patients and their families with a wide range of types of financial resources and (2) conduct ongoing assessment of the financial needs of cancer patients.

Prior studies have demonstrated the efficacy of FN in reducing FT and improving cost-savings for healthcare organizations, yet there is a need to better understand organizational readiness for implementation of FN interventions in diverse settings, including in rural oncology care settings (14, 16–18). Organizations' ability to adequately address patient financial needs is directly related to staff capacity and resource availability within an oncology practice, and rurality of oncology practices often influences the resources and staffing available to that practice. Patients living in rural areas may need additional support from oncology practice staff as well, since they may face more difficulties with health literacy and are less likely to have access to technology and the internet compared to non-rural patients, which are key barriers to accessing or completing applications for financial assistance (8). Implementing new supportive care interventions, like FN, requires additional resources such as dedicated staff, time, and training that may not be available to all cancer care organizations. Selecting and tailoring implementation strategies to support FN that are responsive to the needs of the organization has high face-validity and has the potential to improve practice, though empirical evidence on the efficacy of this approach is lacking (19). It is therefore critical to rigorously assess factors that may support or impede FN implementation in both rural and non-rural settings to guide tailoring of implementation strategies to understand how these approaches can optimize implementation and patient outcomes. This includes attention to administrative burdens, which are defined as the learning, compliance, and psychological costs of program implementation (20).

Nine cancer care clinics were selected to participate in the scale-up and expansion of a novel FN intervention initially tested in a single academic medical center. Lessening the Impact of Financial Toxicity (LIFT) is a multi-site, single-arm trial that aims to examine the impact of FN on experiences of financial toxicity in adult patients with cancer across rural and non-rural settings (21). The FN intervention is designed to have trained financial navigators at each site systematically identify patients at high risk for FT, assess their eligibility for resources, and work with patients and caregivers to help manage their cancer costs. Sites were recruited based on their prior involvement with a statewide survivorship support network, with an emphasis on geographic diversity across the rural-urban continuum. The purpose of this study was to conduct a pre-implementation evaluation of clinic staff perceptions of organizational readiness and barriers and facilitators to successful implementation of a novel FN intervention across rural and non-rural cancer care organizations. We also compared differences between perspectives of staff at rural and non-rural sites.

## Materials and methods

### Setting, sample, recruitment, and study design

To assess rural and non-rural differences, we stratified participating sites by rural status, as defined by the United States Department of Agriculture (USDA) Rural-Urban Commuting Area (RUCA) codes (22). Of the nine sites recruited to participate in LIFT, five were designated rural (22). Key clinic staff, including medical directors, nurse navigators, social workers, and/or financial counselors, from each site were recruited to participate in the preparatory phase focused on understanding readiness and preparedness for FN implementation. We integrated strategies in our recruitment process to maximize the information gathered from all relevant site-level staff who were involved to better inform adaptations of the intervention. We recruited and conducted interviews over a 1-year period to maximize the number of cancer center staff that could be interviewed about the financial navigation intervention. Purposive and snowball sampling approaches were used for recruitment, whereby initial interview participants were purposefully targeted for recruitment based upon their named role at the cancer center (e.g., director, nurse navigator oncology social workers), and those initial interviewees identified other relevant people in their center directly involved in providing financial assistance support in less formal roles. Staff were recruited for surveys and interviews via e-mail and provided with a $50 gift card incentive for their participation.

### Data collection and measures

We used a sequential, mixed-methods approach to meet the aims of this study. Surveys were used to perform an initial, quantitative assessment of organizational readiness. Each staff member completed an individual survey which included demographic questions and site questions along with the Organizational Readiness for Implementing Change (ORIC) scale (23) to assess their perceptions of organizational readiness (Appendix 1). ORIC assesses two facets of readiness: change commitment (organizational members' resolution to implementing a change) and change efficacy (members' belief in the capacity of the organization to undertake a change) (23). The scale has been shown to have high reliability, with alpha coefficients for the Change Commitment Scale and Change Efficacy Scale of 0.91 and 0.89, respectively (23).

Then we recruited staff members who had completed surveys for semi-structured interviews to qualitatively assess readiness and perceptions of barriers and facilitators for implementation, based upon initial interview guides piloted in an earlier iteration of the intervention (24). Two study team members with prior training and experience in qualitative interviewing (MM and MG) conducted 30–45 min semi-structured, in-depth interviews with staff members via phone or a secure video-conferencing platform between April 2020 and April 2021. The interview guide questions were developed using the Consolidated Framework for Implementation Research (CFIR) and assessed stakeholder's perceptions of barriers and facilitators to successful implementation of the FN intervention (Appendix 2) (25). The CFIR is an implementation evaluation framework comprised of 39 constructs nested within 5 domains (inner setting, outer setting, individual characteristics, intervention characteristics, and implementation process) (25). Interviews were recorded and transcribed verbatim.

### Analysis

We calculated descriptive statistics for ORIC survey responses and qualitatively assessed differences between rural and non-rural respondents. Chi-square tests were used to examine statistically significant differences in proportions of agreement with ORIC statements between rural and non-rural participants. However, given the small samples of rural and non-rural participants, we caution against overinterpreting these findings; they are intended to be hypothesis generating only and point to opportunities for future work with more robust samples of rural and non-rural sites. We used a deductive approach to perform a thematic analysis of qualitative interviews, with a codebook informed by the CFIR (25).

For training purposes, six coders (CB, VP, MM, MG, NP, LS) coded three transcripts, and discussed discrepancies to refine codebook definitions. The remaining transcripts were independently coded, with any coding questions resolved within coding team discussions. Transcripts were coded using Dedoose, multifunctional qualitative data management and analysis software (version 9.0.15) (26). Two study team members (CB, VP) identified themes within the coded excerpts from each site. Themes relating to barriers and facilitators to implementation were mapped onto domains of the CFIR. We compared the presence and salience of themes, by rural vs. non-rural site, using a consensus-based, data-driven approach based upon the frequency of themes coded across CFIR domains within rural and non-rural site interviews. Any disagreements in thematic synthesis were resolved through team discussion and review of the raw and coded data. The study team conducted report backs at each site to allow participants to provide input and any further clarification on the findings. No changes to the findings from these analyses were made after the report backs, as the participants agreed with the thematic salience and overall findings from the study. We utilized the Consolidated Criteria for Reporting Qualitative Research (COREQ) to guide reporting of the qualitative findings in this manuscript (27).

## Results

### Sample

Participant characteristics stratified by rurality are provided in Table 1. We approached 84 cancer center staff to complete the ORIC scale and an interview. The ORIC scale was completed by 78 staff members (92.9% survey response rate). Rural site staff members comprised 60.3% of the sample and an average of 8.7 staff per site completed the scale. Of the 78 participants who completed the survey, 73 (55.6% from rural sites) responded to our request for an interview, with an average of 8.1 interviews per site. Five staff members, across multiple sites, who completed the survey were unable to participate in an interview due to scheduling conflicts (*n* = 2) or staff turnover (*n* = 3).

**Table 1:** Stakeholder characteristics, stratified by rurality.

	Overall (*N* = 78)	Rural (*N* = 47)	Non-rural (*N* = 31)
Role^a^			
Administrator/leadership	26 (33%)	17 (36%)	9 (29%)
Oncology nurse navigator	13 (17%)	7 (15%)	6 (19%)
Financial counselor	13 (17%)	6 (13%)	7 (23%)
Registered nurse	11 (14%)	8 (17%)	3 (10%)
Social worker	10 (13%)	5 (11%)	5 (16%)
Lay navigator	3 (4%)	1 (2%)	2 (6%)
Pharmacist	2 (3%)	2 (4%)	0 (0%)
Medical oncologist	1 (1%)	0 (0%)	1 (3%)
Radiation oncologist	1 (1%)	1 (2%)	0 (0%)
Other^b^	13 (17%)	9 (19%)	4 (13%)
Number of cancer patients seen in past week			
**Mean, SD**	**13.8** (**28.7)**	**15.1** (**32.1)**	**11.8** (**23.1)**
0 (not in a clinical role)	47 (60%)	29 (62%)	18 (58%)
≤15	11 (14%)	4 (9%)	7 (23%)
16–25	9 (12%)	7 (15%)	2 (6%)
>25	11 (14%)	7 (15%)	4 (13%)
Years of experience			
**Mean, SD**	**9.3** (**8.2)**	**10.1** (**9)**	**8** (**6.7)**
≤2	15 (21%)	9 (20%)	6 (21%)
3–5	18 (25%)	10 (22%)	8 (29%)
6–10	19 (26%)	12 (27%)	7 (25%)
>10	21 (29%)	14 (31%)	7 (25%)
Years in role at current institution			
**Mean, SD**	**5.6** (**5.4)**	**5.7** (**5.5)**	**5.5** (**5.4)**
≤2	27 (37%)	17 (38%)	10 (36%)
3–5	18 (25%)	10 (22%)	8 (29%)
6–10	20 (27%)	13 (29%)	7 (25%)
>10	8 (11%)	5 (11%)	3 (11%)

SD, standard deviation.

^a^ As it pertains to patients with cancer; participants could select multiple roles as applicable.

^b^ Other includes research nurse, pharmacy technician, oncology coordinator, radiation therapist, patient financial services, nurse care manager, care coordinator, population health navigator.

### Rural and non-rural differences in the organizational readiness for implementing change scale

A comparison of rural and non-rural ORIC results can be found in Table 2. Over 90% of participants across rural and non-rural sites strongly agreed with 9 out of the 12 statements within the Change Efficacy and Change Commitment scales in ORIC, reflecting strong perceptions of organizational readiness. The vast majority of participants across both rural and non-rural sites (88%) reported confidence in their ability to “handle challenges that might arise in implementing this change”. Slightly fewer participants from non-rural sites reported confidence in their ability to manage the politics of change (87% rural, 76% non-rural) and in their organization's ability to support people adjusting to change (96% rural, 75% non-rural). Agreement with the latter statement was statistically significantly different between rural and non-rural sites.

**Table 2:** Perceptions of organizational readiness for Implementing change (ORIC).

People who work here…	Overall	Rural	Non-rural
	(*n* = 78)	(*n* = 47)	(*n* = 31)
Change efficacy scale^a^	% (*n*/*N*)^b^	% (*n*/*N*)^b^	% (*n*/*N*)^b^
…feel confident that the organization can get people invested in implementing this change	92% (70/76)	94% (44/47)	90% (26/29)
… are committed to implementing this change	92% (69/75)	93% (43/46)	90% (26/29)
…feel confident that they can keep track of progress in implementing this change	93% (71/76)	94% (44/47)	93% (27/29)
…will do whatever it takes to implement this change	95% (71/75)	96% (44/46)	93% (27/29)
…feel confident that the organization can support people as they adjust to this change*	88% (66/75)	96% (45/47)*	75% (21/28)*
…want to implement this change	92% (69/75)	93% (43/46)	90% (26/29)
…feel confident that they can keep the momentum going in implementing this change	91% (68/75)	91% (42/46)	90% (26/29)
Change commitment scale^a^			
…feel confident that they can handle the challenges that might arise in implementing this change	88% (67/76)	89% (42/47)	86% (25/29)
…are determined to implement this change	92% (69/75)	93% (43/46)	90% (26/29)
…feel confident that they can coordinate tasks so that implementation goes smoothly	91% (68/75)	91% (42/46)	90% (26/29)
…are motivated to implement this change	95% (72/76)	94% (44/47)	97% (28/29)
…feel confident that they can manage the politics of implementing this change	83% (62/75)	87% (40/46)	76% (22/29)

^a^ Scaled responses dichotomized into “agree/somewhat agree” and “disagree/somewhat disagree/neither agree nor disagree”; percentages reflect percent agreement with the statement.

^b^ Missing values excluded from percentage calculation.

* *p* < 0.05.

### Staff perceptions of barriers and facilitators to implementation

Key themes identified in semi-structured interviews with participants can be found in Table 3. Staff at both rural and non-rural oncology sites highlighted factors reflective of the Intervention Characteristics (relative advantage) and Implementation Climate (compatibility and tension for change) domains as facilitators for successful implementation. The primary differences in perceptions between participants from rural and non-rural sites are highlighted by the barriers identified. On the whole, participants from rural sites did not discuss many perceived barriers to implementation, but when they did, they mentioned factors that reflected staffing and resource constraints. Participants from non-rural sites, on the other hand, identified potential barriers due to the organizational culture and structural characteristics. These findings will be further expanded upon in the sections that follow.

**Table 3:** Key themes identified in stakeholder interviews on barriers and facilitators to Implementation.

CFIR construct	Theme	Barrier or facilitator	Rural, non-rural, both
Intervention characteristics			
**Relative advantage**	Financial benefit to organization	F	Both
	Justify value of FN position to administration	F	Both
	Opportunities to network and share information with other organizations	F	Non-rural
	Fewer delays in treatment, missed or rescheduled appointments	F	Both
	Additional resources, education, and assistance for patients	F	Both
	Confidence in staff's ability to meet financial needs of patients	F	Rural
**Relative advantage and complexity**	Simplification of current processes of financial assistance	F	Rural
**Intervention source**	Prior relationship with study team	F	Rural
Inner setting			
**Implementation climate**			
Compatibility and tension for change	Alignment with organization and staff priorities of addressing financial needs	F	Both
	Awareness of relationship between financial toxicity and cancer outcomes	F	Non-rural
Compatibility	Lack of existing formal FN programs	F	Both
	Lack of adequate staff to address financial needs	F	Rural
	Flexible work environments	F	Both
	Existing work-flows and processes	B	Non-rural
**Readiness**	Competing staff demands and tasks	B	Both
	Physical space to accommodate new staff member/position	B	Rural
	Delegating job responsibilities	B	Rural
	Resources to hire new FN if the position becomes vacant	B	Rural
**Culture**	Resistance to change in work processes	B	Non-rural
**Structural characteristics**	Difficulty communicating changes in processes due to large size of organization	B	Non-rural
	Physical separation of cancer center from main hospital	F	Non-rural
Outer setting			
**Patient needs and resources**	Limited broadband access, low literacy	B	Both
	Privacy concerns, emotional and informational burden	B	Both

## Intervention characteristics

### Relative advantage

#### Relative advantage for the organization

Participants from both rural and non-rural sites discussed how the FN intervention has the potential to provide a financial benefit to the hospital by increasing revenue through insurance maximization and reduced unreimbursed charges. Several staff mentioned that documenting such a benefit would help to justify the value of the intervention to administration in hopes of sustaining a FN program in the long term. Additionally, a non-rural stakeholder specifically mentioned how the intervention would provide opportunities to network and share information with other cancer care centers.

#### Relative advantage for patients

Staff generally perceived that the positive impact of the intervention on patients would facilitate implementation and intervention success.

“*I know that the doctors would probably go for it more than anyone just because, you know, it*’*s hard when a patient is in your face and crying and telling you like they have all this other stuff that they’re stressed and worried about and like they can*’*t focus on themselves and take care of themselves. So I think if they–if the doctors knew that there was such a program or such a process that the patients will be taken care of, everybody can focus more on the patient versus, you know, all this other stuff.”* (Site 9, Non-rural)

They believed that patients would appreciate having extra resources, education and assistance navigating the cost of cancer care from the intervention. They also believed that the intervention would result in fewer delays in treatment and missed or rescheduled appointments.

“*So, I think it*’*s going to be, this position would be, very, very pivotal to our patient population, specifically [county] because we are low income—you know, low literacy level in general, as a county, this—this one of the lowest. So, I think having that person to be that resource to these patients in a time where it's very unknown for them what the future looks like…having someone to say—you know, we*’*re here to help, it*’*ll be okay*.” (Site 5, Rural)

#### Relative advantage specific to rural sites

Participants from rural sites particularly emphasized that the additional knowledge and resources provided by the intervention would give patients confidence in staff's ability to meet their financial needs.

“*I think the hospital is going to embrace this and I think the patients will too because it looks like we have a plan. We are in control. We know how they could get help with their finances, you know?*” (Site 2, Rural)

Several participants from rural sites also mentioned that having a single point person to handle financial needs for patients would be particularly beneficial as it would help facilitate the process of assisting patients follow-through and complete financial assistance applications. Stakeholders perceived that this characteristic of the intervention, also reflective of the ***complexity*** of the intervention, would simplify current processes and help increase organizational capacity to meet patients' needs.

### Intervention source

Few participants mentioned facilitators related to the intervention source. However, stakeholders from a rural site noted that a prior relationship with the research team and university where the team was located provided confidence in implementation of the intervention.

## Inner setting

### Implementation climate

#### Compatibility with organizational culture and values

Participants at both rural and non-rural sites discussed how the intervention purpose was in alignment with organization and staff priorities to assist patients with their financial distress, which garnered support for implementation among staff and leadership.

“*…You want to help your patients and you know how stressful it can be for them. So anything that we can do to help, I think you see that we*’*ll jump on board with it.”* (Site 1, Rural)

“*I think our mission and values play a big part in our wanting to help, and our compassion towards our patients, and doesn*’*t matter where they come from, what they have, what they don*’*t have. We want to help any of them*.” (Site 2, Rural)

In non-rural sites, participants specifically mentioned a cultural awareness among staff of the relationship between financial toxicity and cancer care disparities. These factors were reflective of high ***tension for change*** as they demonstrated shared receptivity to the intervention and a high relative priority for addressing financial needs for cancer patients.

#### Compatibility with existing workflows

Stakeholders discussed how implementation would be facilitated by the lack of existing formal FN programs because there were no practice roles that would conflict with the intervention. Participants also expressed desire for additional structure to the processes currently in place to assist patients with financial needs as many felt that assisting patients with financial needs was a task spread across many staff members rather than a formalized role. Participants perceived that current processes of connecting patients to financial assistance would be bolstered by the intervention by providing additional training, personnel and resources that would increase organizational capacity to meet patient needs.

*“I think [connecting patients with financial assistance is] something that we say is important and I think we all feel that it*’*s important, but I think it*’*s not followed up on like it could be or should be. And some of that is time related, and you get busy and then its, who*’*s taking care of this? Or who*’*s following up on this?”* (Site 6, Non-Rural)

Participants also perceived that the knowledge and resourcefulness of current staff would help facilitate implementation because their knowledge of available resources for cancer patients would supplement the support and resources of the intervention.

#### Rural and non-rural differences in perceptions of compatibility

When discussing how the FN intervention would provide structure to current processes, participants from rural sites described lacking adequate staff to address financial distress in cancer patients in their current processes. Therefore, the lack of existing staff was seen as an opportunity to bring a financial navigator, with a clearly defined role, into the organization. For rural sites, which had less staff, this points to the ***relative advantage*** of the Financial Navigator intervention.

*“I think that you know, especially here, when patients are in the clinic, it seems like things are–it*’*s such a fast pace that sometimes I feel like I don*’*t have enough–I don*’*t want I don*’*t have enough time, but I don*’*t feel like I have enough time to adequately talk to the patient without feeling rushed….”* (Site 4, Rural)

Both rural and non-rural participants also mentioned that they perceived their work environments to be flexible to the addition of a new intervention, but reasons differed by rurality. Participants from rural sites mentioned that their staff would likely adapt to the novelty of financial navigation in their sites, in part, due to the flexible workplace environments and norms they had developed amid the COVID pandemic.

“*Well I think implementing it is—since we*’*re very new and we*’*ve never really had this type of program in the past and [Financial Navigator] is fairly new to this—I think implementing is actually going to definitely help us because we don*’*t have the old* ‘*This is how we do it*’*…it's kind of like,* ‘*Please help us do this. Show us what is the right way to do this.*’” (Site 1, Rural)

Conversely, a non-rural site attributed their flexibility and adaptation to new processes to the physical separation of the cancer center from the main hospital. This separation (between the cancer center and the main hospital) gave staff more liberty to establish new processes and interventions (also reflective of the ***structural characteristics*** of the non-rural site). However, some non-rural participants saw the existence of current processes within their organization as a potential barrier because they were unsure of how well the intervention would be incorporated or how it might interfere with those processes.

### Resource readiness

Both rural and non-rural staff spoke to competing staff demands and high administrative burden of connecting patients to financial assistance as possible barriers to implementation. These factors are reflective of available resources, such as time, that may affect readiness for implementation. One participant mentioned they were concerned about the effects of the intervention being minimized because staff are so often pulled in many different directions. Another participant discussed how implementing new workflows may be difficult with current staff since they are already heavily burdened with tasks and have very little time to complete them.

*“…everybody*’*s spread into so many different places that, maybe, I would hope that it wouldn*’*t get kind of diluted and sort of undermined in a little bit of a way, because there are so many other places they*’*re being pulled.”* (Site 9, Non-Rural)

#### Factors affecting readiness for rural sites

Participants from rural sites expressed concerns about not having sufficient time and physical space within their organization to support the intervention. One participant in particular expressed concern about their ability to utilize an existing employee for the FN position, which would require transferring their job responsibilities elsewhere.

“*Are they going to allow me to hire another social worker, or are they–to cover those hours? Or would they–are they going to say,* ‘*Well, the grant is not going to cover a social work salary. You could have–we could have done this for a lower salary,*’ *if that makes sense*.” (Site 3, Rural)

Another participant expressed concern about the ability to adapt if the financial navigator position became vacant due to the limited additional resources at the hospital to fill such a gap.

### Cultural resistance to change in non-rural sites

Participants from non-rural sites mentioned perceiving general resistance to change within the culture of their organization, facing push-back from providers about a new process, and that other staff members were very rigid in their job responsibilities. One participant expressed this concern while talking generally about implementation of a new role that would require other staff members to initiate a referral to a FN, which, while seemingly minor, still required additional work on top of existing job responsibilities.

“*So I know my personal stance working with some of our financial counselors or our financial like our Medicaid specialists and stuff, they*’*re much like* ‘*I just am helping with the Medicaid application and then that*’*s it.*’ *You know, like hands off for everything else. And so I think sometimes that can be a little frustrating…*” (Site 7, Non-Rural)

### Structural characteristics of non-rural sites

Participants from non-rural sites also pointed to the difficulty of keeping providers and staff informed about the intervention due to the large size of their institution.

“*And the thing that comes to my mind is like, there are just so many people like getting like providers and staff, like getting them all to know about it and to operate the same playbook.*” (Site 9, Non-Rural)

## Outer setting

### Patient needs and resources

Participants from both rural and non-rural sites mentioned that patient needs and resources, such as limited broadband access and low literacy levels, may serve as a barrier to the timely completion of applications for financial assistance resources. Participants also mentioned factors such as patient concerns about privacy or the emotional and informational burden of dealing with a cancer diagnosis as possible barriers to having patients be willing to participate in a FN program.

“*The only thing I could see as a challenge is when they*’*re coming in for us for the new cancer diagnosis, it's just overwhelming and it*’*s just information overload… so many appointments, they meet with the surgeon and get information and then the medical oncologist to get information, and it*’*s just so much.*” (Site 1, Rural)

### Sustainability concerns for rural sites

Although it is not a specific domain of CFIR, when speaking about the sustainability of the intervention, one participant from a rural site mentioned that they were concerned about the potential for patient load to fluctuate in the long term and therefore, the financial navigator might not have enough work. Another rural participant noted the concern about how the position would be funded after the grant period was over.

“*I’m always thinking, okay, what are the longer-term implications too, because actually if you implement something and you want to fully understand exactly what it accomplished here and is that something that we continue to fund and continue to go with? You know, it*’*s very disappointing if you see something that really works, and you*’*re not able to continue to fund it.*” (Site 4, Rural)

## Discussion

Addressing FT among patients with cancer may be enhanced by the implementation of a FN program that includes proactive screening for financial hardship, comprehensive assessment of financial status and vulnerability, linkage to resources, and continued follow-up throughout and beyond treatment. In this study, we interviewed key staff at rural and non-rural cancer care delivery organizations prior to implementation of a novel FN intervention to assess their organizational readiness and perceived barriers and facilitators to successful FN implementation. Results from these assessments and engagement with staff at each site allowed us to tailor implementation strategies to address perceived challenges among organizations with a high degree of willingness and readiness to implement FN.

Participants identified numerous facilitators to implementation that aligned with CFIR domains and constructs, including Inner Setting, Outer Setting, and Intervention Characteristics (25). Participants also identified a handful of potential barriers to successful implementation that aligned with the Inner and Outer Setting CFIR domains. Perceptions of readiness for implementation were strong across rural and non-rural sites. Few differences in perceptions of organizational readiness, barriers and facilitators emerged between staff from rural and non-rural sites; however, rural staff more frequently discussed time demands and resources as barriers to successful implementation while non-rural staff discussed the compatibility of the intervention with existing work processes and resistance to change as barriers. Additionally, it is notable that despite such overwhelmingly high perceptions of readiness measured using ORIC, the primary barriers identified by rural sites (time and physical space resources to support implementation) reflect components of readiness that were not well-captured by the ORIC.

Assessing pre-implementation perceptions of barriers and facilitators and organizational readiness can help guide the refinement of intervention implementation strategies. Tailored implementation strategies can improve implementation outcomes, and theoretically, patient outcomes, if appropriately matched to the needs and context of the organization (19). As a result of this work, we made several modifications to our planned FN implementation strategies to address existing barriers and capitalize on the identified facilitators. First, all results from staff interviews were reported back to each site, discussed, and adaptations made where possible depending on site needs (for example, adaptations to the timing and frequency of financial stress screening were made as a result of workflow and patient needs considerations that came up during interviews). Second, the study team made modifications to the training process to address concerns about role clarity, to facilitate ease of access to training modules and materials given competing demands of staff, and to promote networking and resource sharing among sites. The training was modified to include three longer, instead of five shorter, training sessions with the financial navigators, and trainings were offered remotely and recorded and archived for booster trainings. The team also incorporated one-on-one time with a project manager and tutorial materials so the navigators could practice using the case management database (RedCAP) before starting to recruit patients. During the training process the team created bonding opportunities for the navigators to facilitate connections and networking opportunities between sites, such as icebreakers at the start of training calls. Third, the study team is hosting monthly all-site peer learning collaborative calls for financial navigators to discuss any problems arising during implementation and potential solutions. These calls function as peer professional support for sometimes isolated financial navigators in smaller rural practices and allow the study team and other financial navigators to share new financial resources with each other and brainstorm solutions and workarounds to addressing challenges. Fourth, the study team is holding monthly technical assistance/coaching calls with individual sites for financial navigators to have additional one-on-one time problem-solving and case management check-ins with the study team. Study team members are coding these discussions for alignment with CFIR domains and constructs.

The study team plans to conduct post-implementation patient surveys and key staff member interviews to understand additional experiences and implementation determinants. This will fill a gap in the current literature on FN implementation and add to the empirical evidence on how tailored implementation strategies impact implementation and patient outcomes, particularly in rural vs. non-rural cancer clinical contexts (28). We will also use post-implementation results to inform our assessment of challenges that arise during FN implementation and compare to pre-implementation perceptions.

### Limitations

These results are limited to the key staff members who completed the ORIC survey and participated in interviews. Results from the ORIC survey and interview questions about potential barriers to implementation may have been biased by social desirability since staff were surveyed and interviewed by a study team that was providing financial support to the organization to implement the FN intervention. Additionally, survey responses were dichotomized and limited options to indicate agreement with the ORIC statements limited our ability to distinguish nuances in the strength of agreement. However, our interviews allowed us to obtain more in-depth information on staff member perspectives on implementation and contextualize the findings from the ORIC survey. We also assessed readiness and barriers and facilitators among staff at organizations that had already agreed and were willing to implement the FN intervention. Other organization employees and patients may have had different perspectives on barriers and facilitators to the intervention that were not reported in these results. We did not evaluate differences in perceptions of barriers to implementation by staff role; however, this is an opportunity for future work. Additionally, all intervention sites are located within one state, North Carolina; therefore, reports of rural/non-rural differences in barriers and facilitators may not be generalizable to other settings. That said, North Carolina is a large, geographically, and racially diverse state (44 of its 100 counties are rural, and 29% of its population identifies as persons of color); therefore, the lessons learned in preparing for the implementation of a multisite FN intervention in this state may be helpful for others grappling with how best to address cancer-related financial hardship in diverse patients and settings.

Future directions include evaluations of the differential administrative burdens (20) associated with the implementation of financial navigation interventions in rural and non-rural cancer care settings; specifically, the learning costs (the process of determining eligibility for participation in the FN intervention), the compliance costs (e.g., paperwork associated with enrollment, follow-up; for patients, the required financial documentation to prove eligibility), and the psychological costs (for staff, the distress associated with patients' financial distress; and for patients, the stress, stigma, and shame associated with recognizing and acting upon the need to address cancer-related financial toxicity). This would add to a growing body of work addressing administrative burdens in healthcare (20, 29), which likely interact with the differential travel burdens borne by rural-dwelling cancer patients (30) to shape treatment choices.

Financial navigation is an effective and critical intervention for addressing financial toxicity in patients with cancer that must be adapted and scaled widely to address the pressing material, psychosocial, and health burden caused by the rising costs of cancer care. Pre-implementation interviews with key staff members at rural and non-rural sites demonstrated strong readiness and several notable barriers and facilitators to implementation. These findings informed the tailoring of implementation strategies to support implementation at each site. Post-implementation assessments are necessary to understand how effectively tailored strategies addressed barriers identified by stakeholders—such as resistance to change, competing work demands, and lack of role clarity and resources to accommodate financial navigation—and to identify new barriers that arise during implementation.

## Data Availability

The data are not available due to privacy/ethical restrictions. Requests to access the data should be directed to stephanie_wheeler@unc.edu.
