# Supplementary material for: Assessing the pre-implementation context for financial navigation in rural and non-rural oncology clinics
Source: Front Health Serv. 2023 Oct 23;3:1148887. doi: 10.3389/frhs.2023.1148887 (PMC10627810; doi:10.3389/frhs.2023.1148887)
Supplement: Supplementary file 1 [file Table1.docx]

**Appendix I. Organizational Readiness for Implementing Change (ORIC) Scale**

| 1 | 2 | 3 | 4 | 5 |
| --- | --- | --- | --- | --- |
| Disagree | Somewhat  Disagree | Neither Agree nor Disagree | Somewhat  Agree | Agree |

| 1. People who work here feel confident that the organization can get people invested in implementing this change. | 1 2 3 4 5 |
| --- | --- |
| 1. People who work here are committed to implementing this change. | 1 2 3 4 5 |
| 1. People who work here feel confident that they can keep track of progress in implementing this change. | 1 2 3 4 5 |
| 1. People who work here will do whatever it takes to implement this change. | 1 2 3 4 5 |
| 1. People who work here feel confident that the organization can support people as they adjust to this change. | 1 2 3 4 5 |
| 1. People who work here want to implement this change. | 1 2 3 4 5 |
| 1. People who work here feel confident that they can keep the momentum going in implementing this change. | 1 2 3 4 5 |
| 1. People who work here feel confident that they can handle the challenges that might arise in implementing this change. | 1 2 3 4 5 |
| 1. People who work here are determined to implement this change. | 1 2 3 4 5 |
| 1. People who work here feel confident that they can coordinate tasks so that implementation goes smoothly. | 1 2 3 4 5 |
| 1. People who work here are motivated to implement this change. | 1 2 3 4 5 |
| 1. People who work here feel confident that they can manage the politics of implementing this change. | 1 2 3 4 5 |

**Appendix II. Cancer Center Staff Semi-Structured Interview Guide**

**Introduction:**

Thank you for your interest in this study. Thanks so much for completing the online survey we sent prior to this interview. The aim of this interview is to help us understand how your practice responds to patients with financial problems. Your responses to both the survey and the interview will help us understand how to improve cancer programs’ ability to respond to patients with financial problems moving forward.

We expect that our discussion will last about 45 minutes to an hour. Are you in a place where you feel comfortable and like you can speak freely, i.e., in an office with a door that closes? Everything you tell us will remain confidential and will only be reported as part of a bigger group, without your name attached to it. Before we begin, I would like to ask your permission to audio record our discussion (for research and training purposes). Would it be OK with you if I record this interview? (*If participant refuses to be audio-recorded, the project coordinator will take notes instead*) The interview will be turned into written notes, but your name or any identifying details will not be associated with any of the notes. The audio recordings will be erased once the project is complete.

Do you have any questions before we begin?

If you have any questions, please reach out to the study staff or the PI for this study.

1. Role in the organization
2. I’d like to start by asking you what might happen if I were a patient in your cancer program, **and I asked for help with finances related to my cancer treatment.**

*Depending on the extent to which the interviewee responds to the question above and gives a clear sense of the context of the cancer program as it relates to financial counseling, including determinants of whether and how services are offered, you may also choose to ask the following questions:]*

***Prompts:***

- 1. *What happens when a patient mentions having trouble paying for* ***personal expenses*** *(e.g., rent, electricity, gas) due to the costs of their treatment?*

1. What would happen if I were a patient in your cancer program, and I needed help with my finances related to my cancer treatment, **but I didn’t ask anyone for help?**

***Prompts:***

- 1. *Are all patients asked about their financial assistance needs? Who is responsible for asking patients about their financial assistance needs?*
  2. *What happens when a patient is having trouble paying for personal expenses (e.g., rent, electricity, gas) due to the costs of their treatment?*

1. Now, I’d like to **discuss potential barriers and facilitators** to implementing a specific program to help patients deal with their financial issues in your organization.

The FN program consists of: (1) identification of cancer patients at high risk for, or currently experiencing financial difficulties related to their cancer treatment; (2) connecting these patients to a dedicated oncology financial navigator in your organization (supported by a UNC grant in this context), who will use a comprehensive assessment tool to determine financial needs and one-on-one appointments to direct patients to specific financial support resources and assist with applications; and (3) routine tracking and monitoring of patients’ financial and health outcomes. The patients referred to a financial navigator will have at least 2 visits with the navigator with some patients receiving more intensive, needs-dependent support.

Could you please talk about how implementing an intervention like this in your organization might work? Are there things that would facilitate the intervention’s implementation? What about things that might make implementing the intervention challenging?

**Prompts**

| **CFIR: OUTER SETTING** | |
| --- | --- |
| ***patient needs and resources*** | - How do you think the individuals served by your organization will respond to the intervention? - What barriers will the individuals served by your organization face to participating in the intervention |
| ***Cosmopolitanism*** | - Do you exchange information with others outside of your organization regarding helping patients deal with financial issues? - What professional networking do you engage in? Local or national conferences? Social media? |
| ***Peer pressure*** | - Can you tell me what you know about any other organizations that have implemented the intervention or other similar programs? - To what extent would implementing the intervention provide an advantage for your organization compared to other organizations in your area? |
| ***External policy & incentives*** | - How has the COVID -19 crisis impacted your ability to help patients with financial needs? - Are there any local or national guidelines that play a role in whether or not/how you help patients deal with financial issues? - Are there financial incentives provided by your practice or another organization that would influence whether or not/how you help patients deal with financial issues? |
| **CFIR: PROCESS** | |
| ***Engaging*** | - Are there key influential individuals that would affect whether/how patients get help with financial issues? |
| **CFIR INNER SETTING** | |
| ***Structural characteristics*** | - Do you think how your cancer center is organized affects how people in your organization decide whether or not/how to help patients deal with financial issues? (e.g. social architecture, age, maturity, size, or physical layout) |
| ***Networks & communication*** | - How do you typically find out about new information within your organization/practice, such as new initiatives? - How would information about how to help patients deal with financial issues be shared in your practice? |
| ***Culture*** | - Are there any aspects of your organization's culture (general beliefs, values, assumptions that people embrace) that affects whether or not/how you help patients deal with financial issues in your organization? |
| ***Implementation climate*** | - Do you think there is support to change whether/how you help patients deal with financial issues in your practice? - Are there standard work processes and practices regarding helping patients deal with financial issues in your practice? - Does helping patients deal with financial issues conflict with other priorities in your organization? |
| ***CFIR: INDIVIDUAL CHARACTERISTICS /***  ***TDF DOMAINS*** | |
| ***Knowledge (TDF)/ Knowledge & beliefs about the intervention (CFIR)*** | - Can you talk to me about your understanding of the proposed program? |
| ***Beliefs about capabilities (TDF)/ Self-efficacy (CFIR)*** | - How confident are you in rolling out this program to your patients?  +(*Prompts: problems you may encounter/additional expertise or experience needed*) |
| ***Beliefs about consequences (TDF)*** | - Based on what you know so far, do you think this program is going to be helpful to your patients? |
| ***Motivation & goals (TDF)*** | - How important is it to you to help patients deal with financial issues? |
| ***Memory, attention, & decision processes (TDF)*** | - Are there any systems or processes you have worked out for yourself that you always do when working with a patient about financial issues? |
| ***Social Influences (TDF)/ Individual Identification with the organization (CFIR)*** | - How might views or opinions of others, such as colleagues, patients, professional groups, or others in your practice influence whether/how you help patients deal with financial issues? - Is there consensus in the profession about whether/how patients should be helped with financial issues? |
| ***Emotion (TDF)*** | - How might patient emotions such as worry/concern influence whether or not/how you help patients deal with financial issues? |

Thank you so much for talking with us. We would like to get the perspectives of 5-10 people within your organization, who else do you think we should interview within your organization?
